# Forests as a natural seismic metamaterial: Rayleigh wave bandgaps induced by local resonances

**DOI:** 10.1038/srep19238

**Published:** 2016-01-11

**Authors:** Andrea Colombi, Philippe Roux, Sebastien Guenneau, Philippe Gueguen, Richard V. Craster

**Affiliations:** 1 Department of Mathematics, Imperial College London, South Kensington Campus, London; 2 ISTerre, CNRS, Univ. Grenoble Alpes, France, BP 53 38041 Grenoble CEDEX 9; 3 Institut Fresnel-CNRS (UMR 7249), Aix-Marseille Université, 13397 Marseille cedex 20, France

## Abstract

We explore the thesis that resonances in trees result in forests acting as locally resonant metamaterials for Rayleigh surface waves in the geophysics context. A geophysical experiment demonstrates that a Rayleigh wave, propagating in soft sedimentary soil at frequencies lower than 150 Hz, experiences strong attenuation, when interacting with a forest, over two separate large frequency bands. This experiment is interpreted using finite element simulations that demonstrate the observed attenuation is due to bandgaps when the trees are arranged at the sub-wavelength scale with respect to the incident Rayleigh wave. The repetitive bandgaps are generated by the coupling of the successive longitudinal resonances of trees with the vertical component of the Rayleigh wave. For wavelengths down to 5 meters, the resulting bandgaps are remarkably large and strongly attenuating when the acoustic impedance of the trees matches the impedance of the soil. Since longitudinal resonances of a vertical resonator are inversely proportional to its length, a man-made engineered array of resonators that attenuates Rayleigh waves at frequency ≤10 Hz could be designed starting from vertical pillars coupled to the ground with longitudinal resonance ≤10 Hz.

Controlling the propagation of seismic waves to protect critical infrastructure via a seismic invisibility cloak^1^ is of topical interest. Such ideas of wave control originate in electromagnetism where^2^ demonstrated that Veselago’s^3^ negatively refracting lens is realised through so-called metamaterials. These artificially engineered materials, with non-conventional dispersion properties, are now being transplanted into other wave systems. Notably the field of acoustic metamaterials is rapidly developing where exotic dispersion relationships can achieve elastic wave shielding, super resolution, trapping, and cloaking^4^. We show that locally resonant structures, in this case trees, interact with seismic surface (Rayleigh) waves in surprising ways, making a collection of trees, i.e. a forest, the first-ever observed natural metamaterial for elastic waves.

From the structural point of view, a metamaterial is an arrangement of many elementary cells, with sub-wavelength spacing, each containing an inclusion of another material or a cavity that collectively induce non-conventional dispersion properties, e.g., negative refraction. A metamaterial should be distinguished from phononic crystals, that are popular in many sonic and ultrasonic applications, and which are also formed from an ordered lattice of inclusions/cavities, but now on the wavelength scale, typically showing Bragg-scattering induced bandgaps^5^,^6^. This model is not suitable for controlling surface Rayleigh waves at the geophysics scale where the characteristic wavelengths (*λ*) range from a few, to hundreds of, meters for the frequencies relevant to seismic engineering (1 to 50 Hz). A sub-wavelength arrangement of resonators, known as locally resonant elastic metamaterial^7^,^8^,^9^,^10^,^11^,^12^, allows control of waves at a deep sub-wavelength scale (*λ*/2 to *λ*/15) and can be applied to seismic waves. This concept was experimentally demonstrated for elastic plates, where^13^ have achieved quasi-full control of the *A*_0_ Lamb mode using a random cluster of vertical beams in the kHz regime; the same metamaterial can be used for either elastic energy protection and trapping^14^ or directional cloaking^15^.

Previous experiments on geophysical scales using periodic structures, embedded in the ground^16^,^17^ occur at frequencies too high (≫100 Hz) for practical seismic applications. However^18^, recently demonstrated the first metamaterial for surface wave control using vertical sub-wavelength boreholes, in a sedimentary soil, obtaining partial bandgaps and wavefield attenuation for frequency around 50 Hz. The laboratory experiment in the sonic regime with plate and rods of^13^ implies that the attenuation capacity of vertical resonators could exceed those of boreholes. Indeed, the vertical component of Rayleigh waves should couple with resonators analogously to the *A*_0_ mode for plates and, as in^14^,^19^, generate large bandgaps. The anisotropic and nonlinear response of real soils, the wide variety of anthropic and natural sources generating seismic excitation (e.g. natural seismicity and construction activity), and the fundamental differences between waves in plates and seismic surface waves, mean that one cannot simply conduct a frequency scaling of previous work in the kHz range and that the physics does not trivially translate to this new setting (see discussion for further difference/analogies in the supplementary material).

Forests cover a large percentage of the world’s temperate regions and they could represent a widely available natural metamaterial. If one considers surface wave wavelengths, *λ*, from five, to a few tens of meters (frequencies between 10 and 100 Hz on typical sedimentary soils), then trees can be considered as sub-wavelength resonators. The usage of *natural metamaterial* may sound in conflict with the conventional definition of metamaterial as an artificially engineered object. However this concept is reminiscent of early works on negative refracting metamaterials developed to obtain perfect lenses^2^,^20^. The scalability in frequency of physical laws that underlie metamaterials is a well-established concept^21^, and therefore it is understandable that nature could offer examples of metamaterials from the nano to the meso-scale^22^.

## Experimental Results

**Figure 1:**
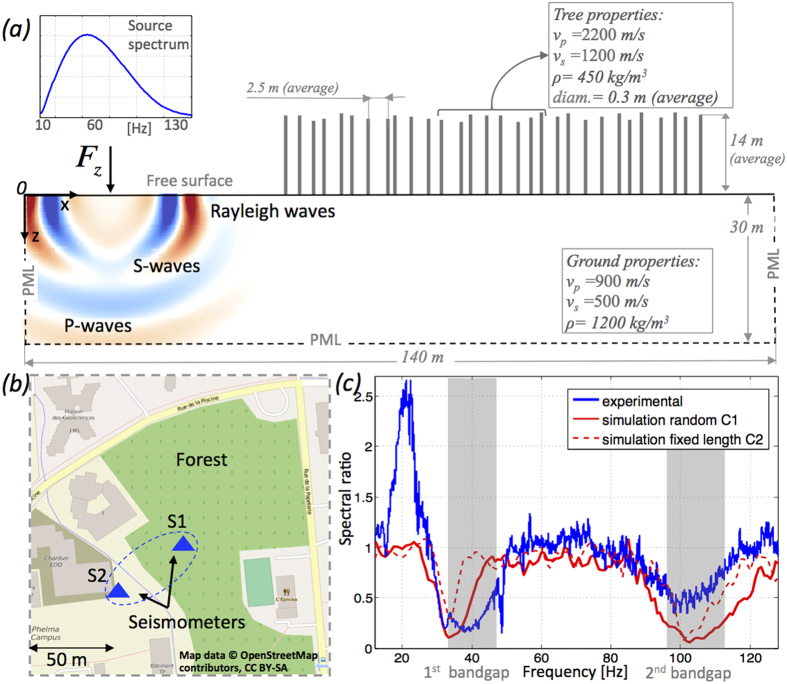
(**a**) The 2D computational domain also showing, in red-blue colorscale, the vertical displacement *u*_*z*_. (**b**) Map (background map from © OpenStreetMaps contributors, https://www.openstreetmap.org/copyright, text and arrows have been added) of the forest location, S1 and S2 are the seismometers. (**c**) Measured (blue) and simulated (red) spectral ratios. Dashed (solid) red line corresponds to the C2 (C1) configuration (see Table 1).

To evaluate tree forests as potential natural metamaterials a geophysical survey on a small forest (~60000 *m*^2^, mainly pine trees) located within the campus of the University of Joseph Fourier in Grenoble was carried out. Two three-component seismometers, S1 and S2, recorded, in continuous mode for 1 hour, the ambient noise inside and outside the region occupied by the forest-metamaterial (see Fig. 1b). The positioning was chosen so S2 was far enough from the forest, whilst S1 was well inside so it can capture the different propagation properties. The total records have been windowed in 10 min records and the averaged spectral ratio between S1 and S2 is depicted in blue in Fig. 1c for the horizontal displacement component *u*_*x*_. Two large transmission minima are clearly distinguishable between 30 and 45 Hz and between 90 and 110 Hz. The energy penetrating through the forest in these frequency bands is strongly attenuated by a factor of 6. It is striking that the second minimum occurs approximately at three times the frequency of the first minimum and hence is related to longitudinal vibrations of the trees^23^. These minima lie in bandgaps created by local resonances between trees and Rayleigh waves propagating in the soil and are explained by extending the beam-plate metamaterial theories of^13^,^19^ and^14^.

The experimental site has a simple geophysical setting: The forest is located on a flat and uniform sedimentary basin. Geophysical surveys show the sediment layer uniformly extends for hundreds of meters down to the bedrock with surface shear velocity varying between 300 and 500 m/s (full details on seismic surveys in the area are given in the supplementary material). The pine trees have straight trunks with length varying between 10 and 20 m and are easily approximated by vertical resonators (hence neglecting the tree crown) given the wavelength under study (*λ* ~ 10 m). They are randomly located with a spacing between 2 and 4 m; the effects of tree roots is negligible because the scattering they produce is of lower order compared to tree resonances (see simulation in the supplementary material). The ambient noise recorded by the seismometers is mainly due to anthropic sources surrounding the experimental area and can be considered locally as omnidirectional. Ambient noise, unlike point source excitation (e.g. shakers), is diffuse because of scattering and reverberations, and it provides a very broad spectrum of frequency excitation. On the day the experiment took place, two particular conditions have been reported. Some construction work on the road contouring the forest on the north side (Fig. 1b) was taking place and moderate wind was blowing making the tree branches move. Either of them could generate some anomalous peak at the seismometer located in the forest (blue curve). On one hand, construction work could cause a local scattering effect on the seismometer outside the forest close to the construction work. On the other hand, wind forces generate flexural tree motion that could convert into particular longitudinal/horizontal excitation at the base of the trees. Note that, with ambient noise excitation, most of the seismic energy propagates as surface waves (Rayleigh and Love^24^ which, at this frequency, have very shallow sensitivity (2–15 m). The elliptically polarised motion of the Rayleigh mode is characterized by a strong, vertical component *u*_*z*_ as shown in Fig. 1a.^13^ and^15^ showed that vertical resonators couple efficiently (eventually creating bandgaps) with this vertical component and so we anticipate that Rayleigh waves will couple with the trees considered as resonators. Love waves, due to their horizontal polarisation, are irrelevant for this study. Forest of trees are therefore a simple model of natural metamaterial for Rayleigh waves characterized by subwavelength bandgaps. As shown in the next section, the variability of soil and tree properties has limited influence on the size and position of bandgaps. Conversely the experiment has shown that bandgaps can be identified using broadband instruments but the measurements may be affected by anthropic sources and experimental conditions.

## Numerical Results

Because of the deep sub-wavelength microstructure of locally resonant metamaterials, it is essential to explore the wavefield within the resonator array with spatio-temporal details that would require thousands of seismometers in the present geophysical configuration. The physics of the sub-wavelength structure is accurately analysed in this paper through time domain spectral element (SEM) simulations. This method has been successfully applied to study the case of a metamaterial plate and rods^14^,^15^. By restricting analysis to Rayleigh waves we reduce the complexity to a 2D halfspace (hence 2D simulations) and consider a linear, isotropic and homogeneous elastic medium with a linear array of 30 trees (Fig. 1a). The simulations are performed with SPECFEM2D^25^. Perfectly matched layer conditions^26^ (PML) are applied on the bottom and vertical boundaries of the halfspace which is otherwise traction-free. The possibility to model the propagation in the 3D locallly-resonant metamaterial with a 2D system has already been demonstrated by solving the eigenvalue problem in the unit cell and it is corroborated by other analytical studies^19^.

The halfspace is characterized by a homogeneous material with shear velocity *v*_*s*_ = 500 m/s and density *ρ*_*g*_ = 1300 kg/m^3^. For a Poisson ratio typical of soil, the Rayleigh wavespeed *v*_*r*_ ~ *v*_*s*_. While these parameters are representative for an average soil, the results discussed next are not strictly limited to this wave speed but they can be generalized to very soft soils featuring *v*_*s*_ < 300 m/s (see supplementary material). The sensitivity of the results to mechanical properties of the soil is therefore limited as long as sedimentary-basins-like soils are considered *v*_*s*_ ≪ 1000 m/s.

Each tree is represented as a homogeneous elastic vertical resonator with constant thickness (hence constant cross-section) characterised by both longitudinal and flexural modes (Fig. SM1 in the supplementary material). We use trees of both constant and random size as well as random spacing between trees to evaluate the effects of the variability that characterizes natural forests; the heights are drawn from a uniform distribution with mean 14 m varying between ±2.5 m. The configurations are labelled as C1-C3 and defined in Table 1. Typical mechanical properties of wood can be found in^27^ and vary widely depending on species, fluid content, and age. From this data, a reasonable approximation, is density, *ρ*_*t*_ of 450 kg/m^3^. Using elastic velocities *v*_*s*_ and *v*_*p*_ shown in Fig. 1a, the Lamé parameters are well within the ranges given for wood.

A sensitivity analysis on the acoustic impedance of the ground 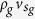 and the trees 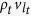 where 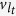 is the tree’s longitudinal wave velocity is performed; parameters given in Fig. 1a are for a unitary ratio between the two impedances and a fluctuation of the ratio between 0.5 and 2 (well within admissible material parameter ranges) results in very similar bandgaps (see supplementary material). For stronger impedance mismatch however, they tend to disappear progressively, confirming the mechanical coupling between ground and trees is the driving parameter. The vertical force in Fig. 1a is driven by a Ricker source time function of dominant frequency 60 Hz^27^. Most body wave energy generated by the vertical force disappears through the bottom boundary, as it should, leaving only horizontally propagating Rayleigh waves (Fig. 1a and video in the supplementary material). The broad spectrum of the source (inset in Fig. 1a) allows us to analyze the wavefield between 10 and 130 Hz. Given *v*_*s*_ = 500 m/s, surface waves have wavelength, *λ*, between 4 and 40 m. A reference simulation of the halfspace without trees is used to compute the spectral ratio, ensuring the red curves in Fig. 1c do not depend on the source spectra and can be compared with the experimental ones between S1 and S2.

**Figure 2:**
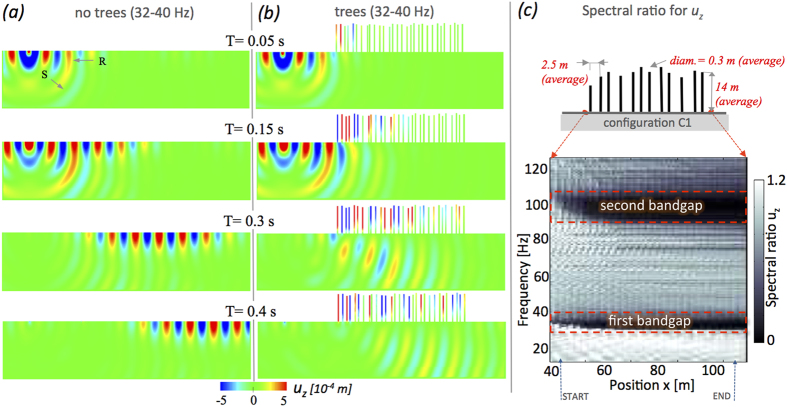
Snapshots at different times for *u*_*z*_, with, (**b**), and without,(**a**), tree filtering in the first bandgap (Fig. 1c); R and S indicate Rayleigh and S-waves. (**c**) Spectral ratio measured through the forest with the start and end points of the array marked on the *x*-axis.

**Figure 3:**
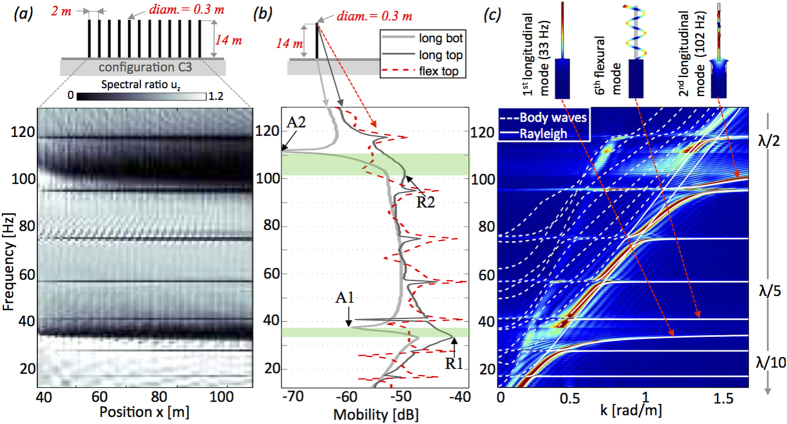
(**a**) Same as Fig. 2c but for resonators of equal size and spacing (C3 in Table 1). (**b**) Frequency response (mobility in dB) of one tree-like resonator for flexural (red) and longitudinal component (dark and light grey). The points where the measures have been taken are highlighted at the top of the panel. (**c**) Dispersion curves computed with Bloch modeling in white superimposed with those from 2D time domain simulations in color scale. Dashed vs. continuous lines distinguish body waves from surface waves. The top of the panel shows the two longitudinal modes responsible for the bandgaps and one flexural mode. The right axis recalls the number of trees per wavelength for the configuration in (**a**).

The interactions between surface waves and trees underlying the experimental results are captured by analyzing Figs 2 and 3. As expected, we observe the so-called hybridization phenomenon^12^,^28^ that drives local resonances and bandgaps in this type of locally-resonant metamaterial. Indeed, the longitudinal resonances of tree-like resonators, excited by the vertical component (*u*_*z*_) of the Rayleigh waves, introduces a phase shift of *π* on the incident waves causing a reflection of the wavefield around the resonant frequencies. At anti-resonance, the point of attachment between ground and tree (*z* = 0, the forcing point) is at rest^19^,^29^ and thus *u*_*z*_ = 0. Because the trees are arranged on a sub-wavelength scale, the cumulative effect of several trees over a wavelength interferes constructively^28^ thus creating a band gap between resonance and anti-resonance. Figure 3 clearly shows that this physical description explains the bandgaps in the Rayleigh wave dispersion curve despite the complexities of the equations of motion. In this physical model, the role played by the narrower flexural resonances of the tree-like resonator is marginal, despite the expected coupling with the Rayleigh wave horizontal component, yet visible with forest made of trees of the same length (further details in the supplementary material).

Returning to the experimental vs. numerical ratios shown in Fig. 1c, there is strong agreement of the overall position of the bandgaps. Given the physical model based on the longitudinal resonance and anti-resonance, the introduction of tree length variability (expected in the forest) produces a curve that better approximates the experimental results (dashed vs. continuous red line). Intuitively the longer the resonator, the lower the first longitudinal resonance and thus bandgaps associated with different tree heights overlap; their presence is averaged in the resulting effective properties of the metamaterial and larger bandgaps are observed.

The motion of the Rayleigh wave is characterized by both *u*_*x*_ and *u*_*z*_, but for brevity and due to the importance of the vertical displacement only *u*_*z*_ is shown The same results and comments apply to *u*_*x*_. In Fig. 2a,b snapshots of *u*_*z*_ with no trees are compared to those from randomly distributed array of trees (C1, Table 1). Both wavefields have been filtered in the first bandgap (32–40 Hz) shown in Fig. 1c. As previously observed, the Rayleigh wave rapidly dominates and propagates toward the tree array. The metamaterial behavior of the forest result in no-surface wave propagating beneath the trees in Fig. 2b. Unlike a plate where the non propagative regime admits only total reflection of the incident waves, in Fig. 2b part of the energy escapes into the bulk with an incidence angle that is the result of the conversion of Rayleigh waves into shear waves and that can be precisely predicted by Snell’s law. This curious phenomenon will be the topic of a future publication. Thus the metamaterial determines the wave propagation properties deep into the halfspace. Using as input the unfiltered (broadband) fields in Fig. 2a,b, in Fig. 2c is depicted the behavior of the spectral ratio as we penetrate deeper, from left to right, into the array of trees. Notice the two bandgaps appearing progressively after a so-called skin layer. As we proceed towards the tail of the array (at *x* ~ 115 m) the bandgap width no longer changes. Because of the absence of a depth dependent velocity gradient, waves diving toward depth do not return to the surface. Hence, the bandgaps persist behind the trees.

Turning now to bandgap width, we notice that longitudinal resonance and anti-resonance frequencies determine start and end points of the bandgap. This is clearly shown by considering an array of equally sized and spaced trees. Figure 3a presents the same spatially dependent ratio as Fig. 2c but with configuration C3. The absence of height, and thickness variability result in two main bandgaps with the same width as the dashed line in Fig. 1c and narrower bandgaps due to flexural resonances.

The frequency response for *u*_*x*_ and *u*_*z*_ in Fig. 3b is computed for a single tree on the halfspace, and exhibits the modes responsible for the bandgaps in Fig. 3a. As expected the longitudinal anti-resonances are most visible at the driving point (*z* = 0) while resonances are most visible at the free-end of the tree^29^. Dark and light grey curves demonstrate that the start and end of the bandgaps are constrained by the R1, R2 and A1, A2 points highlighted in Fig. 3b where R stands for resonance and A for antiresonance; to a larger R-A interval corresponds a larger bandgap.

For a similar metamaterial made of beams fixed to a plate^19^ derived a theoretical relationship based on the scattering matrix method that predicts the position of the frequency R and A. However, their method is limited to the case of the plate and cannot be straightforwardly extended to a resonator attached to the halfspace.

In Fig. 3c we also show the dispersion curves computed using Bloch theory^30^ and COMSOL multiphysics. The eigenmodes are evaluated using a 2 m large elementary cell each containing one tree. Bloch conditions are applied to the two vertical sides of the cell, whilst on the bottom PML is used and traction-free conditions are imposed at the free surface. The graphical representation for the first few eigenmodes is given on the top panel of Fig. 3c (full details in the supplementary material). The white lines, derived using Bloch modeling, are superimposed onto the *f-k* decomposition^31^ computed from the time-domain SEM simulations in Fig. 3a; as expected, the match is perfect. The first and second longitudinal modes generate the two bandgaps, while the several narrow stop bands are due to flexural modes. The dashed and continuous lines distinguish respectively the radiative region where only body waves propagate, from the surface wave region characterised by a lower wave velocity. In the radiative part, a small signature of the *P*-wave dispersion is visible. Finally, the right axis of Fig. 3c gives the average distance between resonators (in wavelength unit) in order to highlight the sub-wavelength character of the metamaterial.

## Discussion

We have, for the first time at the geophysics scale, demonstrated conclusively that a locally resonant metamaterial made of vertical resonators, with dynamic properties similar to those of forest-trees arranged on a sub-wavelength scale, induce large frequency bandgaps for Rayleigh waves at tens of Hz. Seismic records on a forest of trees are interpreted from the cumulative effect of longitudinal resonance inside the trees causing two highly attenuating regions between 15 and 130 Hz. Interestingly, the variability in size and positions of trees produces larger bandgaps than for uniform configuration. Numerical simulations show the extent of the bandgap is governed by the longitudinal resonance and anti-resonance of the tree-like resonator that is further confirmed using Bloch theory. Finally ad-hoc seismic metamaterials for Rayleigh wave attenuation at frequencies ≤10 Hz could be obtained from vertical pillars with longitudinal resonance ≤10 Hz. These results suggest that many applications in electromagnetic metamaterials can be translated to elastic waves. Our study bridges exciting wave phenomena unveiled in photonic metamaterials with surface seismic waves propagation in soils structured on a large scale by natural, or man-made, collections of resonators. A high-resolution geophysical survey on a larger forest supported by a dense acquisition grid (resolution down to 4-m) is planned for next year. We expect that continuous ambient noise as well as controlled source records with a dense spatial sampling will permit to study in great detail the locally-resonant metamaterial behavior of a forest of trees.

## Methods

### Experimental methods

The two three-component seismometers are made by FairField nodal. The multichannel acquisition board (CityShark II) synchronise and digitalise the signals from the seismometers with a sampling frequency of 250 Hz. The one hour long ambient noise records, after being deconvolved from the instrumental response, have been high-pass filtered and windowed in ten-minutes long records. For each trace we have calculated the frequency spectrum and hence the spectral ratio between the two instruments from the synchronous records. The plot in Fig. 1c shows the average spectral ratio for the horizontal component of the motion.

### Numerical methods

The propagation of seismic waves in a 2D halfspace is a well-known problem in numerical seismology and modeled by solving the *P* − *SV* elastic wave equation (*P*: Primary or compressional wave, *SV*: vertically polarized shear or Secondary wave), but its coupling with resonating tree-like elements is rather unusual. The accuracy of the method has been thoroughly tested using plate and rods as input model and it has delivered excellent results. The 2D time domain simulations are carried out using SPECFEM2D a code that solves the elastic wave equation using finite difference in time and the spectral element method in space. The parallelization is implemented through domain decomposition with MPI. The mesh is made of quadrilateral elements and it is generated using the commercial software CUBIT. Simulations are then run on a parallel cluster (Froggy at University of Grenoble) on 16 CPUs. 2D plots have been generated with Paraview.

The dispersion curves in Fig. 3c have been generated solving the eigenvalues problem on the unit cell using the COMSOL Multiphysics software suite with the Matlab interface. The solution is computed via finite element using Bloch-Floquet conditions on the left and the right boundaries of the unit cell.

## Additional Information

**How to cite this article**: Colombi, A. *et al.* Forests as a natural seismic metamaterial: Rayleigh wave bandgaps induced by local resonances. *Sci. Rep.* **6**, 19238; doi: 10.1038/srep19238 (2016).

## Supplementary Material

Supplementary Information

Supplementary video

## Figures and Tables

**Table 1:** Size and spacing of the tree-like resonators for the numerical configurations simulated in Figs 1, 2, 3.

Parameter/configuration	C1	C2	C3
Length [*m*]	12–16	14	14
Thickness [*m*]	0.2–0.4	0.2–0.4	0.3
Spacing [*m*]	1.5–4.0	1.5–4.0	2
