# Supplementary material for: Forests as a natural seismic metamaterial: Rayleigh wave bandgaps induced by local resonances
Source: Sci Rep. 2016 Jan 11;6:19238. doi: 10.1038/srep19238 (PMC4707539; doi:10.1038/srep19238)
Supplement: Supplementary Information [file srep19238-s2.pdf]

SUPPLEMENTARY MATERIAL FOR THE ARTICLE:

Forests as a natural seismic metamaterial: Rayleigh wave bandgaps induced by local resonances

Andrea Colombi, Philippe Roux, Sebastien Guenneau, Philippe Gueguen and Richard V. Craster

SM1 The analogy between a forest-metamaterial and a plate and beam metamaterial

The first results on this sort of locally resonant metamaterial have been obtained for the highly idealized situation of vertical beams fixed to a thin plate in the kHz regime both experimentally (Rupin et al 2014), numerically (Colombi et al 2014) and theoretically (Williams et al 2015).
Limiting the study to the lower branch of the A_0 mode, as in the aforementioned studies, shows the bandgaps emerging as a result of the coupling between the A_0 mode, polarized out of plane, and the longitudinal resonances of the rods (Fig. SM1a). Flexural modes are virtually invisible in the dispersion diagrams (Fig. SM1a), as they do not propagate in the plate (unless the plate gets very thin), this despite their characteristic behavior in the vertical beams. Starting from this observation, Williams et al (2015) have demonstrated that bandgaps in the plate can be studied analytically by considering only longitudinally oscillating resonators and hence via a reduced system of 1D resonators fixed on a 1D oscillating string.

In the more realistic, and more complicated, case of an elastic halfspace, the surface Rayleigh waves are polarized both in and out of plane forcing the base of the tree in a much more complex way than occurs in the case of the plate. Thus, the previous results on plates could not be trivially extended to this new problem without the analysis reported in the main article.
The trees planted on a halfspace (either soft or hard) have both longitudinal and flexural modes (James et al. 2014), but now the latter radiate into the halfspace and both are now visible in the dispersion curves of the metamaterial (numerical results in Fig. 3c). Nevertheless, Fig. 3 chiefly demonstrates that large bandgaps are still created by longitudinal resonances and the flexural ones contribute to very narrow stopbands which are only visible in the dispersion curve if one considers equally parameterized trees (hence configurations C3 in Tab. 1).
Fig SM1b shows only dispersion curves for the low frequency branch, from 0 Hz to the first bandgap and it thus provides a full view of the first modes that characterize the tree-like resonator (configuration C3 in Tab. 1). Full numerical results have been omitted in this plot to ease the interpretation; this plot complements Fig. 3c of the main article.

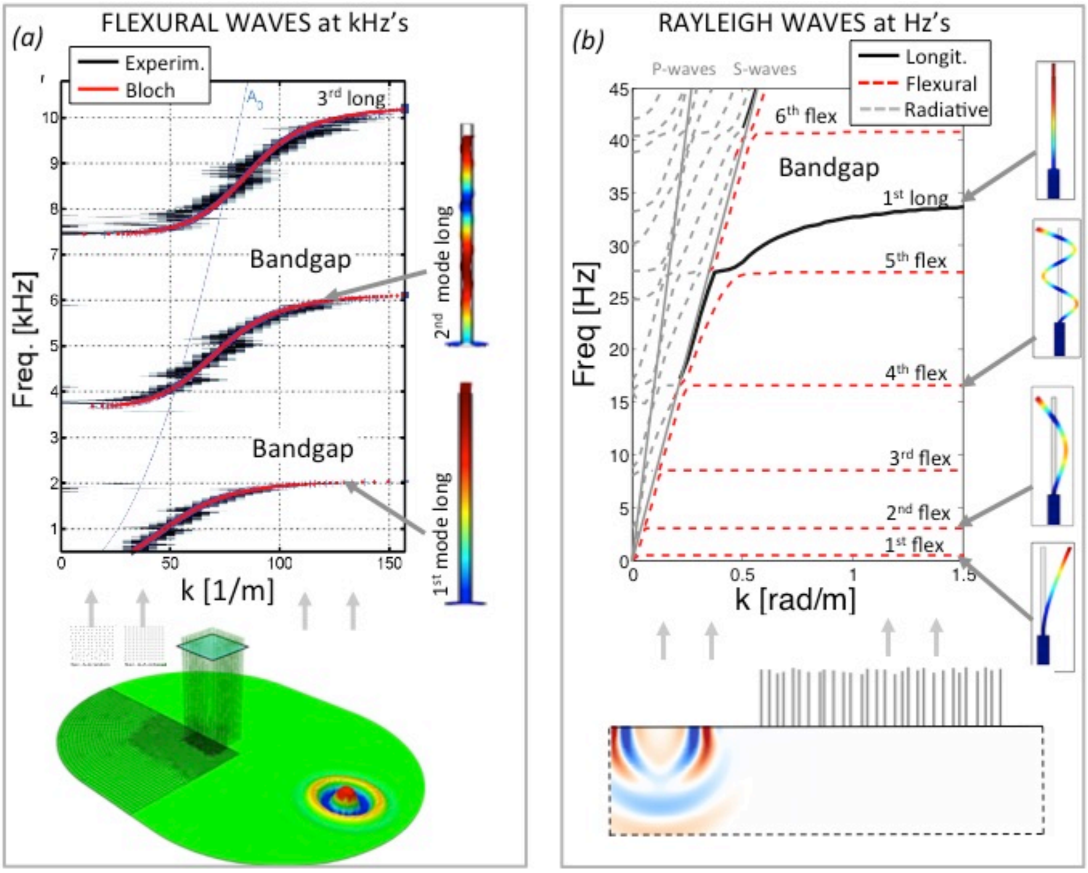

Figure SM1 Comparison between wave propagation physics in the metamaterial made with the plate and with the halfspace. (a) (Adapted from Williams et al (2015) and Colombi et al (2014)) Dispersion curves measured (black) and computed with Bloch theory (red) for the case of the plate. Arrows point to the respective modal deformation. (b) Same as (a) but for the case of the trees on the halfspace. The S-waveline bounds the radiative zone on the left not involved in the analysis.

SM2. Sensitivity analysis over soil parameters.

To demonstrate the robustness of the local resonance against model parameters such as Rayleigh wave velocity and wavelength in the soil, we have computed spectral element simulations (using SPECFEM2D) with several different velocity models.

Fig. SM2 shows the spectral ratio as we penetrate deeper in the array for two soil models. This is analogous to Fig. 2c of the main article where we adopted the random configuration C1. Notice that here we have used a shorter simulation time and therefore the frequency axis is less densely sampled.

In both cases, despite the large changes in Rayleigh wave velocity (close to 700 m/s in Fig. SM2a, and 300 m/s in Fig. SM2b) we only observe small differences with respect to the configuration discussed in Fig 2c of the main article (where the Rayleigh velocity is approx. 500 m/s). Strong variations in the ground material properties slightly modify the eigenmodes of the trees resulting in the small variations in size and position of the bandgaps observed in figure SM2. Some effect at high frequency (>110 Hz) is visible in Fig. SM2a and b, this is probably due to a flexural resonance located very close to the bandgaps creating the impression of a very large bandgap. In addition, for the configuration in FigSM2b the metamaterial at frequency > 110Hz is no longer subwavelength and this may result in different behavior. In a layered soil these results would not change.

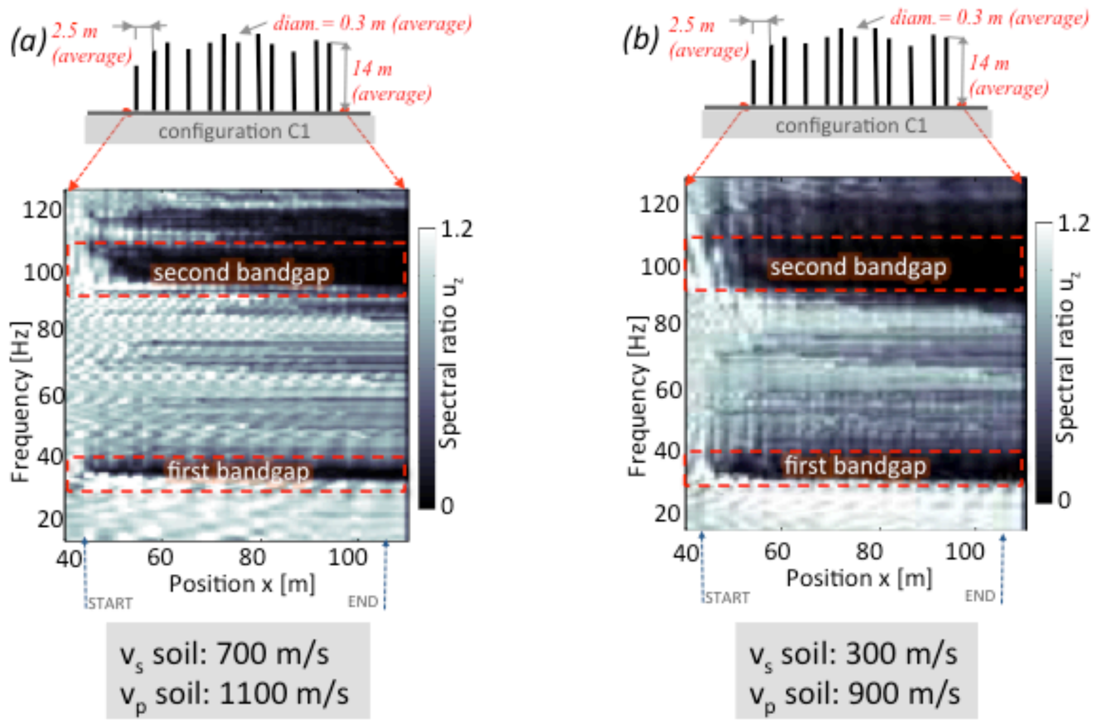

Figure SM2. Same as Fig. 2c in the main article but for different ground velocity models.

Study	Depth z [*m*]	Shear velocity [*m/s*]	Location
Cornou et al. (2004)	0 – baserock	$300 + 1.9\sqrt{z}$	Grenoble basin
Bettig et al. (2001)	0 – 140	350 – 400	University campus & nearby
Gueguen et al. 2007	0 – 4<br>4 – 12	240<br>350	2 km from university campus
Deitrich et al. 2009	4 – 12	400	Northern Grenoble basin

Table SM1 Shear velocity values given from previous study for the Grenoble basin in locations close to the test site.

Shear velocity values for the Grenoble basin are collected in Tab. SM1 and fully justify our choice of model parameters.

From the results of this section, and those discussed in the main article, concerning the impedance mismatch, we conclude that the observed bandgaps are a robust phenomenon with respect to parameter variations within the admissible range for sedimentary soil and trees.

SM3 The effect of roots

To verify whether roots may alter the coupling between trees and soil or not, we have inserted a buried heterogeneity beneath the tree trunk in the numerical model. The heterogeneities are approximately 1.5x1.5 m large and they mimic the presence of roots. The mechanical properties of the heterogeneities results from an average value between soil and trees to take into account that roots

represent rigid inclusions in a soft soil matrix. The value chosen for the roots region are: v_p=1600 m/s; v_s=900, m/s, ρ=1100 kg/m^3.
The background model for trees and soil is presented in Fig. 3a of the main article where the trees have all the same length.
The spectral ratios depicted in Fig. SM3, clearly prove that roots do not influence the presence of the bandgaps. A slight reduction of the spectral coefficients (not observed in Fig. 3a in the main article) appears at frequencies >110. At this frequency range the scattering caused by the heterogeneities could dominate over resonance effects.
We therefore conclude that roots may have an effect over Rayleigh wave propagation, further reducing the amount of elastic energy that penetrates in the array of trees. However this effect seems to be limited to high frequency and of second order compared to resonant effects induced by trees.
To avoid any bias and to ease the interpretation of the resonance phenomena, the results presented in the main article do not consider roots as a model parameter.

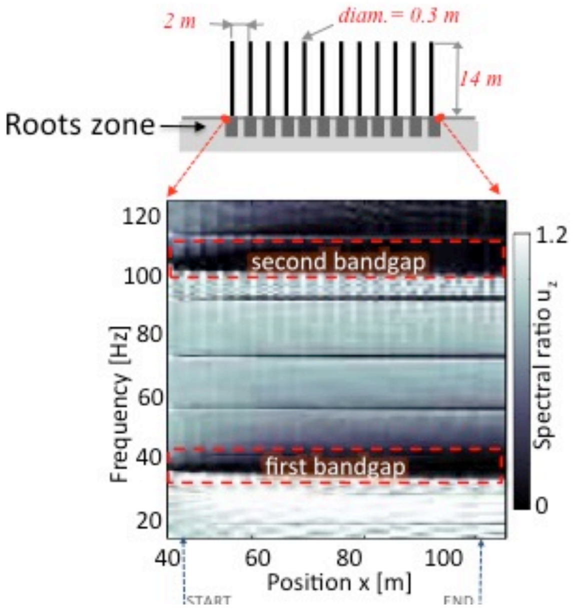

Figure SM3 Configuration used to test the effect of roots on the propagation of Rayleigh waves and the corresponding spectral ratio. The configuration is analogous to that in Fig. 3a in the main article.

Bibilography

Bettig, B., Bard, P. Y., Scherbaum, F., Riepl, J., Cotton, F., Cornou, C., Hatzfeld, D. (2001). Analysis of dense array noise measurements using the modified spatial auto-correlation method (SPAC): application to the Grenoble area. *Bollettino di GeofisicaTeoricaedApplicata*, 42(3-4), 281-304.

Colombi, A., Roux, P., and Rupin, M. (2014) *J. Acoust. Soc. Am.* 135.

Chopra, K., 1995 *Dynamics of structures: theory and applications to earthquake engineering* (Prentice Hall, Englewood Cliffs, NJ) 4th edition.

Cornou, C., Kristek, J., Ohrnberger, M., Di Giulio, G., Schissele, E., Guillier, B. and Moczo, P. (2004, August). Simulation of seismic ambient vibrations: II. H/V and array techniques for real sites. *In Proceedings of the 13th World Conference on Earthquake Engineering*, Vancouver, Canada, August 2004, Paper (Vol. 1130).

Dietrich, M., Cornou, C., Ménard, G., Lemeille, F., Guyoton, F., and Guiguet, R. (2009). Seismic profiling and borehole measurements in the Isère valley near Grenoble, France: 1 data acquisition and processing. In *3rd ESG symposium* ,

Grenoble, 2006

Guéguen, P., Cornou, C., Garambois, S., and Banton, J. (2007). On the limitation of the H/V spectral ratio using seismic noise as an exploration tool: application to the Grenoble valley (France), a small apex ratio basin. *Pure and Applied Geophysics*, *164*(1), 115-134.

James K. R., G A. Dahle, J. Grabosky, B. Kane, and A. Detter. 2014. Tree Biomechanics Literature Review: Dynamics. *Arboriculture & Urban Forestry* 40(1): 1–15

Williams, E.G, Roux, P., Rupin, M., and Kuperman,W. A. (2015)*Phys. Rev. B* 91, 104307.
